# Religious Perspectives on Precision Medicine in Singapore

**DOI:** 10.1007/s41649-021-00180-4

**Published:** 2021-09-06

**Authors:** Hui Jin Toh, Angela Ballantyne, Serene Ai Kiang Ong, Chitra Sankaran, Hung Yong Tay, Malminderjit Singh, Raza Zaidi, Roland Chia, Sarabjeet Singh, Swami Samachittananda, You Guang Shi, Zhixia Tan, Tamra Lysaght

**Affiliations:** 1 Centre for Biomedical Ethics, Yong Loo Lin School of Medicine, National University of Singapore, Singapore; 2 Department of Primary Health Care and General Practice, University of Otago, Wellington, New Zealand; 3 Hindu Centre, Singapore; 4 Taoist Mission (Singapore), Singapore; 5 Sikh Advisory Board, Singapore; 6 Jaafari Muslim Association Singapore, Singapore; 7 National Council of Churches of Singapore, Singapore; 8 Young Sikh Association (Singapore), Singapore; 9 Ramakrishna Mission, Singapore; 10 Singapore Buddhist Federation, Singapore

**Keywords:** Precision medicine, Religious views, Health data sharing, Genomics, Private sector, Health equity

## Abstract

Precision medicine (PM) aims to revolutionise healthcare, but little is known about the role religion and spirituality might play in the ethical discourse about PM. This Perspective reports the outcomes of a knowledge exchange fora with religious authorities in Singapore about data sharing for PM. While the exchange did not identify any foundational religious objections to PM, ethical concerns were raised about the possibility for private industry to profiteer from social resources and the potential for genetic discrimination by private health insurers. According to religious authorities in Singapore, sharing PM data with private industry will require a clear public benefit and robust data governance that incorporates principles of transparency, accountability and oversight.

**Supplementary Information:**

The online version contains supplementary material available at 10.1007/s41649-021-00180-4.

## Background

Governments around the world are investing in large scale national programmes for precision medicine (PM) and Singapore has recently launched the National Precision Medicine (NPM) strategy (PRECISE n.d.). While definitions can vary, PM essentially aims to optimise health outcomes by using knowledge about genomics, lifestyle, and environment factors to better tailor medicine and healthcare (Wang et al. 2016). Unlike the traditional medical practice of prescribing similar treatment plans for patients with common clinical presentations, PM stratifies patients into sub-groups thereby facilitating more precise diagnoses and targeted therapies.

Public attitudes are important for gauging the social licence needed to conduct data-intensive research in the absence of specific consent. PM requires broad public buy-in and cooperation. To understand how genomics, lifestyle, and environment factors interact, researchers need access to substantial amounts of data containing information about clinical observations, genomics and health outcomes from a diverse range of volunteers. Tracking the health of individuals and groups over time requires sophisticated infrastructure to collect, store, link, and re-purpose data for analysis. Given the scope of PM research, multiple parties in both the private and public sectors are typically involved, including public health services, academic researchers, private sector pharmaceutical and biotechnology companies, private insurance companies, and technology companies (Wang et al. 2016). Longitudinal large-scale data collection and sharing across the public and private sector raise several challenges including data security, privacy, public trust, as well as the scope of data-subjects’ consent and social licence for data sharing (Chataway et al. 2012).

This Perspective summarises the outcomes of knowledge exchange in a facilitated workshop with religious authorities in Singapore (see Annex A in the Supplementary Material) to share their perspectives on PM, and data sharing with the organizations in the public and private sectors. To date, there has been little published about the role of religion and spirituality in relation to the ethical challenges of PM (Prosperi et al. 2018; Fisher et al. 2020) or public attitudes to PM (Yeary et al. 2020; Sanderson et al. 2017). Broadly speaking, we know that religious views and practices influence health behaviours and treatment efficacy (Fisher et al. 2020; Ogden 2016) and that religiosity has been associated with a lack of faith in science (Johnson et al. 2015). Sanderson et al. (2017) found that “very religious” respondents in the USA were less willing to participate in biobanking research than non-religious participants; and therefore recommend that PM programmes actively address the concerns of religious communities to ensure diverse representation in PM cohorts. Sheppard et al. (2018) also found that religiosity was associated with lower rates of biospecimen donation for PM-related research.

It is worth noting that this prior research on religion and PM (Yeary et al. 2020; Sanderson et al. 2017; Sheppard et al. 2018), or more broadly on religiosity and views on genetic testing and genomic research (Kinney et al. 2006; Lee et al. 2019), are nearly exclusively based in the USA and may not be reflective of the relationship (if any) between religiosity and views on PM in Singapore or other Asian countries. Therefore, it is important to consider whether PM research and governance processes need to address specific religious considerations in relation to PM in Singapore. Research shows that religious leaders and proscriptions can be influential in their congregations’ behaviours and decisions about health interventions (Thomas et al. 2015; Hungerman 2012). A study in Jordan found that religious permission for biobanking^1^ was one of the two most influential factors for prompting public participation in biobanking (Ahram et al. 2014). For these reasons, we invited religious authorities from across Singapore to share knowledge in a workshop on ethical issues raised by PM. Outcomes from this workshop will be of interest to academics and policymakers responsible for establishing the social licence for PM.

## A Workshop with Religious Authorities in Singapore

This Perspective presents the main outcomes of the workshop. Many of the ethical and governance issues raised during the discussions correspond with prior research on public attitudes towards PM globally (Holden et al. 2019). Here, four broad themes are highlighted: (i) religious values and support for PM in foundational principles, scripture or teaching; (ii) potential religious concerns/challenges for PM; (iii) potential concerns associated with sharing PM data with commercial or private partners; and (iv) recommendations for governance approaches in PM in Singapore. There was no outright disagreement between religious leaders on any points discussed; rather representatives of particular religions choose to speak to certain points, or emphasise particular values or religious tenets.

## Religious Values and Support for PM in Foundational Principles, Scripture or Teaching

The discussions suggested no foundational religious objections to PM or scripture that would directly prevent participation in PM. Rather, the representatives in attendance emphasized values of charity, community, assisting others and acting for the greater good, which were perceived to lend support for participation in PM research. Participating in PM research could be viewed as a way of fulfilling a duty to help those sick or suffering.

*securing public benefit and safeguarding humanity’s well-being, is of utmost importance.* (Muslim representative)

However, equity and access to medicine, specifically the products of PM, remain significant considerations. The increasing cost of medicine is already a concern in Singapore (How and Fock 2014), and many current medical treatments and procedures are widely viewed to be out of the reach of average Singaporeans. This disparity creates inequality between individuals, which is likely to be at odds with some religious teachings. Taoism, for example, is aligned with relational justice through its emphasis on justice as “*dao”* (way-making) (Tan 2019). This implies that all people are of equal worth and, therefore, should have equal access to medicine that potentially helps to save lives.

*all these high cutting-edge medicine which costs is not something which is affordable for most people. In a way, you definitely do [have] discrimination between the haves and the have nots.* (Taoist representative)

## Potential Religious Concerns/Challenges Regarding PM

There was general consensus that people contributing data to PM ought to undergo a robust informed consent process, and that PM data should not be used or shared without consent. Minimally, broad consent is needed at the point of recruitment into the PM programme. Broad consent is an alternative to specific consent and focuses on describing the general purpose and scope of the research programme. Broad consent should include sufficient information to enable a reasonable person to reach a decision about whether to consent or decline to participate; but this need not involve specifying every conceivable research use (Maloy and Bass 2020). These broad consent approaches are typically used in PM because of the difficulty in describing all possible future uses of data at the point of recruitment into a PM programme and the high cost (in terms of time, human resources and funding) of concurrently seeking consent for each data use (Schaefer et al. 2019). Under the Singaporean Human Biomedical Research Act 2015, broad consent can be obtained in lieu of specific consent for storage and secondary uses of identifiable data and biospecimens (Goh 2018). It is becoming generally accepted that genomic data cannot be truly anonymised (Shabani and Marelli 2019). The consent process for participants entering a PM programme of research must therefore include information about who the data may be shared with, the process of de-identification, and the potential risk of re-identification, even where this risk is small.

More specifically, the representatives from Catholicism and Hinduism emphasized rationality, free will, autonomy and personal responsibility. The following Catholic teaching demonstrating the relationship between morality, responsibility and voluntariness: “Freedom characterizes properly human acts. It makes the human being responsible for acts of which he is the voluntary agent. His deliberate acts properly belong to him” (Catholic Church 2000, 1745). Hinduism also places importance on the individual and individual will. For example, Hindu rituals and practises are diverse with significant freedom for individual choice and preference (Harman 2004); and Hindu ethical teaching focuses on the “cultivation of the self” (Baird 1998) towards the goal of non-attachment.

*as far as Hinduism is concerned, it gives a lot of importance to the individual and individual will* (Hindu representative)

From these perspectives, the absence of specific consent to each instance of data sharing in a PM programme could be seen as a potential barrier to individuals’ ability to make decisions and responsibly exercise their free will. The value of individual autonomy and responsibility in Catholicism and Hinduism is in prima facie conflict with broad-, blanket- and future-consent models in PM. Yet, representatives recognized that medical technology and genomic research methods develop; therefore, not all future uses of PM data could reasonably be predicted at the time of enrolment in a PM programme. Some suggested that, in order to respect individual autonomy, periodic re-consent may be preferable to relying solely on broad consent at the time of enrolling in the programme. During the re-consenting process, significant changes in the scope of sharing, linking and new uses of the data should be communicated to participants in a PM programme.

Representatives suggested that using data outside the parameters of the individual’s consent would raise moral challenges. In the absence of specific consent, transparency and the right to withdraw from a PM programme were seen as important mechanisms for promoting autonomy and free will as embodied in religious teachings. The ethical need for transparency is closely linked to the value of human autonomy, which allows individuals the opportunity to make decisions independently by weighing information in light of their convictions and values (Felzmann et al. 2019). Transparency may help ensure that participants can access knowledge about how their data is being used and enable them to withdraw from the programme if they believe that data uses are outside the parameters of their original consent or their current expectations.

## Potential Concerns Associated with Sharing Data With Commercial or Private Partners

Representatives at the workshop expressed scepticism towards data sharing with commercial or private partners, including pharmaceutical and biotechnology companies, private insurance companies, and technology companies. As indicated above, charity and community benefit were central pillars for all representatives present at the workshop. While this focus on charity provides impetus to participate in a PM programme that aims to improve community health and wellbeing, they explained that it also underpins a strong reluctance to allow the commercialisation and commodification of PM data, and private profiteering from social resources. Commercialisation and commodification of PM data can be interpreted as treating PM data as “an asset” and selling or distributing PM data for private financial gains (Bottis and Bouchagiar 2018).

*if it’s for commercial use, and for profiteering from the information, that we do not support also. But if it’s for something that medicine to cure that will help humankind, we are for it.* (Taoist representative)

*the most important part is to assure that the personal information or the information obtained will not be used for any other purpose other than people consented for or for commercial gains. While the intent of benefitting humanity is commendable, it needs to be ensured that the use of personal data for purposes other than service to humanity will not happen.* (Muslim representative)

International empirical research has consistently demonstrated that the public and patients are reluctant to allow the use of health-related data for commercial purposes (Shabani et al. 2014; Hill et al. 2013; Kalkman et al. 2019; Grant et al. 2013). The NICE Citizens Council (2015) in the UK found that half of the Council had concerns about anonymous data^2^ being shared widely, and these related in part to objections to data being sold on to other organisations, used for profit, or for commercial purposes other than research. In response, some data access policies limit access by commercial entities (Shabani and Borry 2016). There was concern expressed at the workshop that sharing PM data with commercial companies could also facilitate genetic discrimination by insurance companies, and inequity arising from the development of expensive and inaccessible drugs by pharmaceutical companies (Lee et al. 2019).

From a Catholic perspective, health may be viewed as a fundamental right. Pope Benedict XVI has been emphatic on the need to “guarantee adequate [health] care to all” (Sibley 2016). As such, health should not be left to the market to determine or distribute.

*Health is a fundamental right. It’s not like they’re offering other kinds of things that are optional for people.* (Catholic representative)

This reasoning shares commonalities with the work of bioethicists Norman Daniels (1985) who has written about the role of health as a primary good. Daniels uses the Rawlsian (Rawls 1999) framework of primary goods and fair equality of opportunity to draw attention to the specific importance of health care. Daniels argues that health care deserves special attention because of the unequal natural distribution of health care needs and because of the strategic importance of health to an individual’s opportunity range and future life choices. On this view, justice requires that institutions ensure that the *social basis of health* should be distributed fairly within the community. This means that access to the benefits of social institutions that affect people’s ability to protect and maintain their health, arguably including PM programmes, should be fairly distributed amongst the community. It was reasoned at the workshop that for-profit companies and commercial interests in PM should not be permitted to distort or unfairly determine access to health resources.

Nonetheless, there was some openness to Public Private Partnerships (PPPs) or data-sharing arrangements that produce public benefit in addition to private profit. For example, Catholicism accepts the role of private property, commercial interests and economic role of markets, so long as these are arranged to serve the higher purposes of allowing individuals to express their free will and aligned with the common good and communal purpose (Koterski 2012).

*there is nothing wrong with profit. [If] the profit is done by a private company for the common good, they deserve that profit.* (Catholic representative)

PPPs can be an effective method of harnessing the potential of health and genomic data and can include public and private sector partners working across the data chain—producing, analysing, using or repurposing data (Ballantyne and Cameron 2019). Programmes such as the Innovative Medicines Initiative’s (IMI) established by the European Union works as an international data sharing platform aiming to stimulate novel drug development. Some of the most successful biobanks, such as UK Biobank (n.d.) and FinnGen (n.d.), allow commercial and private sector organisations access to their data. Religious representatives involved in this workshop expressed openness to public private partnerships or data-sharing arrangements with commercial companies that produce public benefit in addition to private profit.

## Recommendations for Governance Approaches in PM

The general consensus at the workshop was that a commitment to public benefit, transparency, accountability and robust governance models will be necessary to engender the support of religious authorities for the sharing of PM data from Singapore with commercial companies, especially in the absence of specific consent for such sharing. Trustworthy governance should include a review process for data sharing arrangements to ensure the public benefit requirement is met. This review process should include lay perspectives. Existing oversight and approval mechanisms for health data sharing include Data Access Committees (DACs) and Institutional Review Boards (IRB).^3^ However, in Singapore, IRB approval would not be required for sharing de-identified/non-personal PM data with research partners, including private industry. The Personal Data Protection Act (PDPA) and the Human Biomedical Research Act (HBRA) apply only to personal (reasonably identifiable) data sharing. Workshop discussions concluded that, given the national interests in protecting and stewarding Singaporean genomic data, establishing a national PM oversight body or panel responsible for making decisions about when to share PM data, particularly if private industry is involved, may help to engender trust within religious communities about data sharing arrangements.

There was also consensus about data sharing being guided by the principles of openness, transparency, honesty and accountability. These norms were seen as essential pillars for a trustworthy and religiously acceptable PM programme. Openness — with respect to ownership of the data, users of the data, place of storage, and rights retained by the donors of the data — would demonstrate respect to those who contribute to PM:

*As far as the Christian faith is concerned, it does come back to the dignity of the human being who has contributed those data.* (Christian representative)

To further facilitate transparency and accountability, an audit trail for the data sharing should be publicly available. The outcomes of discussions suggested that this openness includes a list of all PM projects, including collaborations or public–private partnerships using the data. The UK Biobank (n.d.) takes this approach: projects using the data are described on their website. Obligations of transparency may conflict with commercial confidentiality with respect to some public–private partnerships; nonetheless representatives stressed that transparency is a high priority for ensuring moral legitimacy and public trust. The data governance principles raised throughout the conversations, included accountability, transparency, and dignity/respect, align with those described elsewhere (Merson et al. 2015; Xafis et al. 2020) and will be an important component of data governance structures for PM.

## Conclusions

This Perspective describes the outcomes of a workshop for knowledge exchange between representatives of the major religions in Singapore on PM and sharing data with public agencies and commercial companies. The general consensus to emerge from the workshop was that PM could be an important contributor to the common good that should be embedded with values of charity, community, respect and trust. Securing the social license would thus require robust and trustworthy systems of governance and oversight to ensure transparency, accountability and the equitable distributions of benefits. This consensus accords with the bioethical principles and norms identified in the prior theoretical and empirical literature on health data governance.

## Supplementary Information

Below is the link to the electronic supplementary material.

Supplementary file1 (DOCX 32.6 KB)

## Footnotes

1 Some representatives were shown a statement indicating that religion does not prohibit individuals from donating biospecimens for research and biobanking.

2 There is growing consensus that true anonymity of data is now technically impossible; therefore, we prefer to talk about degrees of data de-identification and security rather than anonymous data.

3 DACs are a group that is charged with the responsibility of reviewing and assessing data access requests; they have a dual responsibility with respect to “both promotion of data sharing and protection of data subjects, their communities, data producers, their institutions and the scientific enterprise” (Cheah and Piasecki 2020). IRBs (in some jurisdictions these are called Research Ethics Committees) are charged with reviewing and approving human subjects research.
