# Supplementary material for: Religious Perspectives on Precision Medicine in Singapore
Source: Asian Bioeth Rev. 2021 Sep 6;13(4):473–83. doi: 10.1007/s41649-021-00180-4 (PMC8486897; doi:10.1007/s41649-021-00180-4)
Supplement: Supplementary file 1 — Supplementary file1 (DOCX 32.6 KB) [file 41649_2021_180_MOESM1_ESM.docx]

**Annex A: List of workshop participants**

| No. | Organization | Designation |
| --- | --- | --- |
| 1 | Singapore Buddhist Federation | Chairman of General Affairs |
| 2 | National Council of Churches of Singapore | Lecturer in Systematic Theology, Trinity Theological College |
| 3 | National Council of Churches of Singapore | Lecturer in Systematic Theology, Trinity Theological College |
| 4 | Catholic Church of Singapore | Priest/Spiritual Director |
| 5 | Caritas Singapore | Special Projects Executive |
| 6 | Catholic Medical Guild | Immediate Past Master, Catholic Medical Guild |
| 7 | Ramakrishna Mission | President |
| 8 | Hindu Centre | Committee Member (Research & Publications) |
| 9 | Hindu Centre | Advisor to Education Committee and Hinduism Teacher |
| 10 | Jaafari Muslim Association Singapore | President |
| 11 | Jaafari Muslim Association Singapore | Honorary Secretariat |
| 12 | Islamic Religious Council of Singapore (Muis) | Senior Executive, Office of the Mufti (Research) |
| 13 | Islamic Religious Council of Singapore (Muis) | Executive, Office of the Mufti (Religious Policy) |
| 14 | Sikh Advisory Board | Chairman |
| 15 | Young Sikh Association (Singapore) | President |
| 16 | Sikh Welfare Council | Vice Chairman |
| 17 | Taoist Mission (Singapore) | Secretary-General |
| 18 | Taoist Mission (Singapore) | Vice President |
| 19 | Taoist Federation | Priest |

**Annex B: Workshop handout**

**HANDOUT: PRECISION MEDICINE RELIGIOUS WORKSHOP**

**Thursday 1^st^ October 4.00pm - 5.30pm**

**Host:** Prof. Tamra Lysaght (Centre for Biomedical Ethics, National University of Singapore)

**Questions:** Please contact Toh Hui Jin [medthj@nus.edu.sg](mailto:medthj@nus.edu.sg) [+65- 9172 4814].

The purpose of this workshop is to help us better understand what perspectives, if any, religious authorities in Singapore have about precision medicine, specifically with respect to data-sharing with private industry and commercial partners.

We aim to address the following questions during the workshop, time permitting:

- Are there any concerns in general with a programme that gathers genome sequence data from your followers, links it to their electronic medical record and de-identifies it for use in research (with their consent)?
- What, if anything, would you advise your followers if they were to ask about being recruiting onto this type of programme?
- What, if any, value do you see in such a programme?
- Are there any concerns you would have about de-identified data from your followers being shared with private industry for precision medicine? Private industry may include:
  - Pharmaceutical and biotech companies
  - Tech companies (e.g. Google)
  - Private health insurers
- What, if anything, would you advise your followers if they were to ask about being recruited onto a programme that will allow private industry/commercial partners to access the data?
- What, if any, value do you see in sharing health data with private industry?
